# Parasitemia and Differential Tissue Tropism in Mice Infected with *Trypanosoma cruzi* Isolates Obtained from *Meccus phyllosoma* in the State of Oaxaca, Mexico

**DOI:** 10.3390/pathogens11101141

**Published:** 2022-10-02

**Authors:** Any Laura Flores-Villegas, Jesús Guillermo Jiménez-Cortés, James González, Adriana Moreno-Rodríguez, Rebeca Pérez-Cabeza de Vaca, Claudia Segal-Kischinevzky, Martha I. Bucio-Torres, José A. De Fuentes-Vicente, Elisabeth Nava-Lazaro, Paz María Salazar-Schettino, Margarita Cabrera Bravo

**Affiliations:** 1 Departamento de Microbiología y Parasitología, Facultad de Medicina, Universidad Nacional Autónoma de México, Avenida Universidad 3000, Ciudad Universitaria, Coyoacán, Mexico City 04510, Mexico; 2 Departamento de Biología Celular, Facultad de Ciencias, Universidad Nacional Autónoma de México. Avenida Universidad 3000, Ciudad Universitaria, Coyoacán, Mexico City 04510, Mexico; 3 Laboratorio 16, Facultad de Ciencias Químicas, Universidad Autónoma “Benito Juárez” de Oaxaca, Avenida Universidad S/N, Ex Hacienda de Cinco Señores, Oaxaca 68120, Mexico; 4 Centro Médico Nacional “20 de Noviembre”, ISSSTE, Félix Cuevas 540, Del Valle, Benito Juárez, Mexico City 03229, Mexico; 5 Tecnológico de Monterrey, Escuela de Medicina y Ciencias de la Salud and The Institute for Obesity Research, Monterrey 64710, Mexico; 6 Instituto de Ciencias Biológicas, Universidad de Ciencias y Artes de Chiapas, Libramiento Norte Poniente 1150, Tuxtla Gutiérrez 29050, Mexico

**Keywords:** *Trypanosoma cruzi*, *Meccus phyllosoma*, parasitemia, tropism, *tssa* expression, virulence

## Abstract

*Trypanosoma cruzi* is a parasite transmitted by the feces of triatomines. Many triatomine species are found in Mexico, and various *T. cruzi* variants have been isolated from these species, each showing very different virulence and cell tropism. The isolates were obtained from *Meccus phyllosoma* specimens in three localities in the state of Oaxaca, Mexico: Tehuantitla, Vixhana, and Guichivere. The virulence of each isolate was assessed by quantifying parasitemia, survival, and histopathologic findings. The lineage of each isolate was identified using the mini-exon gene. The expression of the *tssa* gene during infection was detected in the heart, esophagus, gastrocnemius, and brain. Our results show that the maximum post-infection parasitemia was higher for the Tehuantitla isolate. On genotyping, all isolates were identified as *T. cruzi* I. The amastigotes in the heart and gastrocnemius were verified for all isolates, but in the brain only for Tehuantitla and Vixhana. The *tssa* expression allowed us to detect *T. cruzi* isolates, for Tehuantitla, predominantly in the heart. For Vixhana, a higher *tssa* expression was detected in gastrocnemius, and for Guichivere, it was higher in the esophagus. Results show that virulence, tropism, and *tssa* expression can vary, even when the isolates are derived from the same vector species, in the same region, and at similar altitudes.

## 1. Introduction

Chagas disease is a significant zoonosis caused by the flagellate parasite *Trypanosoma cruzi*, transmitted by hematophagous insects of the subfamily Triatominae (Hemiptera: Reduviidae). This disease is endemic in Latin America; an estimated 6–8 million people worldwide are infected, and the parasite causes approximately 50 thousand deaths annually [1]. Chagas disease is regarded as a public health problem in Mexico, where an estimated 4.06 million people are infected, and 29.5 million are at risk of infection [2]. The most affected age group is 25–44. The Mexican states with the highest number of reported acute cases are: Veracruz, Jalisco, Morelos, Querétaro, Oaxaca, Chiapas, and Guerrero. In 1940, Mazzotti described the first cases of the disease in the state of Oaxaca. Currently, a Chagas disease prevalence of 17.7% has been estimated in the region, with 672,947 infected individuals [3]. In localities of the Isthmus of Tehuantepec, where 11 species of triatomines are known to be vectors of the disease [4], autochthonous cases of *T. cruzi*-associated cardiomyopathy, megaesophagus, and megacolon have been reported [5].

Mexico is one of the Latin American countries that harbors the most significant number of vector species for Chagas disease, with more than 34 triatomine species. Among these, the six species of the *Meccus* complex, *Meccus pallidipennis*, *M. longipennis*, *M picturatus*, *M. mazzottii*, *M. phyllosomus*, and *M. bassolsae*, are important as vectors of *T. cruzi*. These species are located in wild ecotopes and are often found inside homes in areas with high entomological indices [6]. Due to their morphology and phylogenetic closeness, this species complex was subjected to revalidation, and the *Triatoma phyllosoma* complex was reassigned to the genus *Meccus* [7,8,9]. The six species in the genus *Meccus* are distributed in almost 50% of Mexican states; they are estimated to be involved in three out of four cases of vector-borne transmission of *T. cruzi*.

The presence of *M. phyllosoma* (Burmeister, 1835) has been reported in the state of Oaxaca, Mexico, located at an altitude between 10 and 1200 meters above sea level (masl) and is considered a significant vector for *T. cruzi* transmission in the Isthmus of Tehuantepec [10,11,12]. The biological importance of this species lies in its ability to form hybrids with other species of the same genus, which increases the risk of *T. cruzi* transmission [13].

A genetically diverse parasite, *T. cruzi*, is currently classified into seven discrete typing units (DTU), termed as TcI to TcVI and Tcbat. Their differences in behavior and virulence can be attributed to the interaction with factors such as the environment, the host, and the vector [14]. Parameters such as the prepatent period, mortality/survival rates, parasitemia, and tropism to target organs have been measured to determine the isolates’ virulence. As such parameters are tested in animal models, virulence varies with the susceptibility of the experimental model, the host immune system, the inoculum type and size, and the isolate [15]. In addition, abiotic factors such as altitude are known to play a role in the virulence of isolates, as shown by studies with the vectors *Triatoma dimidiata* and *T. barberi* [16,17].

A molecular marker widely used for *T. cruzi* identification is the *tssa* (trypomastigote small surface antigen). This protein is expressed on the surface of trypomastigotes, playing a pivotal role in parasite infectivity and differentiation. In addition, the *tssa* overexpression helps the morphological transformation from trypomastigote to amastigote. The *tssa* has been proposed as a good candidate for molecular diagnostics or the development of Chagas disease vaccines [18].

The virulence of three *T. cruzi* isolates from *M. phyllosoma* specimens collected in Oaxaca, Mexico, was measured herein. Parasitemia levels and survival rates were quantified in a murine model. In addition, histopathological studies were performed, and the lineage of each isolate was molecularly identified using a mini-exon as a reference, and the *tssa* expression was detected on different days of infection to determine tissue tropism on heart, esophagus, gastrocnemius, and brain.

## 2. Material and Methods

### 2.1. Origin of Isolates

**Figure 1:**
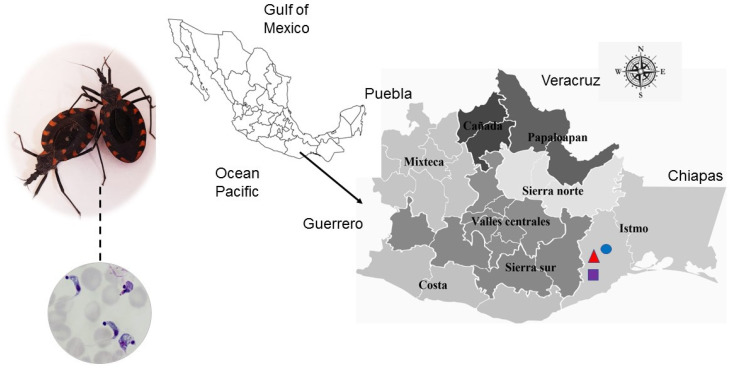
*T. cruzi* isolates (**dotted line**) obtained from the droppings of naturally infected *M. phyllosoma* specimens in the state of Oaxaca, Mexico. The map depicts the eight socioeconomic regions of the state; the localities under study were Tehuantitla (**triangle**), Vixhana (**circle**), and Guichivere (**square**) in the Isthmus of Tehuantepec.

The isolates used herein were obtained from the droppings of *T. cruzi*-infected *M. phyllosoma* triatomine specimens from the state of Oaxaca, Mexico (Figure 1). The insects were collected in the localities of Tehuantitla, Vixhana, and Guichivere, near the Isthmus of Tehuantepec region. Isolates were established in a murine model by two cyclic passages and named in accordance with the nomenclature proposed by the WHO (Table 1) [19].

### 2.2. Parasitemia

For each isolate, 48 female CD-1 mice weighing 18–22 g were used. Thirty-two mice were randomly selected and infected with each *T. cruzi* isolate (*N* = 32) (four replicate, eight mice per replicate), in addition to a control group of 16 animals (*N* = 16) (four replicate, four mice per replicate). A total of 48 animals was used in each isolate. Infected mice were intraperitoneally inoculated with 10^6^ parasites in 1 mL [20]. The control group was inoculated with sodium citrate (3.8%) by the same route. Every other day, a blood sample was taken from the distal end of the tail of mice. The number of blood trypomastigotes was determined in 10 µL of collected blood diluted 1:10 with sodium citrate (3.8%) in a Neubauer chamber, under an optical microscope (40×). Parasitemia was quantified for 30 days or until mouse death, and the number of parasites/mL was reported.

### 2.3. Survival

Daily death/survival events were recorded in infected and control mice for 30 days. The survival rate is defined as the number of mice that persisted to the end of the experiment (30 days). The median survival is the day on which 50% of mouse mortality occurred [21]. Euthanasia in a CO_2_ chamber was applied, considering the presence of signs such as palpebral ptosis, bristly hair, and hindquarter involvement. Euthanasia was performed in accordance with NOM-62-ZOO-1999 “Technical specifications for the production, care and use of laboratory animals”. The appropriate Ethics Committee approved this study with permit No. 001-2020/004-CIC-2020.

### 2.4. Organ Harvesting

Animal deaths were verified by the absence of corneal reflex and rhythmic respiration after euthanasia in a CO_2_ chamber. For dissection, the subjects were placed on a wax base and secured for a longitudinal cut from the stomach to the pharynx. The heart and esophagus were identified for removal; the gastrocnemius was obtained by the dissection of the hind leg. For brain extraction, the skin was removed with toothed dissecting forceps until the external fibrous tissue was exposed. Then, a longitudinal cut was made with curved-blade Mayo scissors and the brain (soft tissue) was removed with a grooved director.

For total RNA extraction, the four organs were obtained from two infected mice and one control mouse on specific days after infection (14, 18, 20, and 22). The organs were collected in 1.5-mL cryotubes, placed on storage rods, and immersed in liquid nitrogen for 30 min. Next, the organs were mechanically disaggregated with sterilized plastic pistils and stored at −20 °C until use.

### 2.5. Histopathological Studies and Amastigote Nest Count

The organs for histopathological study were excised from three mice infected with each isolate, which were euthanized on the day of maximum parasitemia. The organs were placed in Falcon tubes (Sarstedt) with 15-mL of 10% formaldehyde for fixation. Sagittal sections were made in paraffin-embedded organs. Ten 4-micron serial sections were made from each organ and stained with hematoxylin-eosin (H-E) to identify amastigote nests in the heart, esophagus, gastrocnemius, and brain; a total of 100 micro serial sections were made in each organ of each mouse. In each slice, 100 fields were counted under a 100X objective, using a light microscope (Zeiss Primo Star, Oberkochen, Germany) and the number of amastigote nests was recorded. The maximum number of amastigote nests found was taken as 100% [20].

### 2.6. DNA Extraction

Genomic DNA was extracted from each organ using the commercial Dual Genomic DNA Isolation Kit (Tissue Reagent Dual Column Based) (Amerigo Scientific, Hauppauge, NY, USA), following the manufacturer’s instructions (sample preparation, lysis, removal of protein debris, washes, and DNA elution).

### 2.7. Identifying Discrete Typing Units by PCR

Discrete typing units (DTU) were identified by PCR for each isolate. The intergenic region of the mini-exon gene of *T. cruzi* was used for lineage classification. The mini-exon intergenic region was amplified using the oligonucleotides TC1Fw, 5’-GTGTCCGCCGCCACCTCCTTC-3’ or TC2Fw, 5’-CCTGCAGGCACACACGTGTGT-3’ and TCRv, 5’-CCCTCCCCCAGGCCCCACACT-3’ (Souto et al., 1996), to obtain 350-bp (TcI) or 300-bp products (TcII, TcV, and TcVI), or no product (TcIII and TcIV).

A thermocycler and GeneDireX DNA Taq polymerase were used for PCR reactions (10 µL). The PCR program was run as follows: 94 °C for 5 min (1 cycle), 94 °C for 30 s, 55 °C for 30 s and 72 °C for 30 s (30 cycles). The PCR products were stained with GelRed (10X) and separated by 2% agarose gel electrophoresis.

### 2.8. RNA Extraction and cDNA Synthesis

The RNA was extracted by the acid guanidinium thiocyanate—phenol—chloroform reagent method, which was adapted and standardized for 100 mg of tissue. Each tissue sample had 1 mL of TRIzol^®^ reagent added to it (Life Technologies, Carlsbad, CA, USA) and then mechanically homogenized with plastic pistils. The mixture was then transferred to a 1.5-mL tube and incubated for 5 min at room temperature. Then, 200 μL of chloroform per milliliter of TRIzol^®^ were added; the samples were next vortexed and incubated for 3 min at room temperature. After centrifugation at 12,000× *g* for 15 min at 4 °C, the aqueous phase was transferred to a new tube. 0.5 mL of cold isopropanol was then added and allowed to precipitate overnight at −20 °C. The samples then were centrifuged at 12,000× *g* for 10 min at 4 °C, and the supernatant resultant removed. The resultant pellet was washed with 1 mL of ethanol (EtOH, 75%) and centrifuged at 7500× *g* for 5 min (this wash was performed twice). Subsequently, the pellet was washed again with 1 mL of EtOH (100%) and allowed to dry. Finally, total RNA was resuspended in 200 µL of injectable water and stored at −70 °C.

After quantifying and verifying the integrity of the total RNA, 2 µg of the total RNA were used for cDNA synthesis (20 µL). The High-Capacity cDNA Reverse Transcription Kit (Applied Biosystems, Waltham, MA, USA) was used for the cDNA synthesis using RT random primers and following the vendor’s recommendations.

### 2.9. qPCR

A real-time quantitative PCR (qPCR) was performed by the standard curve method with oligonucleotides specific for the *tssa* (ID: AI077116 and AZ049892) and the *actb* (ID: P60710), coding for the antigenic surface adhesion protein of *T. cruzi* trypomastigotes and the abundant cytoskeleton housekeeping protein of β-actin, respectively. The oligonucleotides *tssa* (EMT5/a) 5’-TTTGAGGAGGCTTCTGCT-3’ and the *tssa* (T5/ATG/E) 5’-ATTCATGACTACGTGCCG-3’, which were obtained from the genomic sequence of clone CL Brener as previously described [22,23]; Actb, 5’-TCTGGCACCACACCTTCTAC-3’ and Actb, 5’-TTCACGGTTGGCCTTAGGGT-3’. All oligonucleotides were initially screened for absence of dimers or cross-hybridization. For qPCR, cDNA samples (1:10 dilution) and a Rotor-Gene Q thermal cycler (Qiagen, Hilden, Germany) were used. The SYBR Green dye (2× KAPA SYBR FAST qBioline) was used for detection. The qPCR protocol was as follows: 95 °C for 10 min (1 cycle), 95 °C for 15 s, 58 °C for 30 s, and 72 °C for 30 s (35 cycles). The *tssa* transcript data were normalized to the *actb* transcript amounts. The relative expression change was assessed in terms of the change in induction relative to time zero (uninfected mice). The mean value and standard error of at least two independent biological replicates are reported, where each biological replicate involved four technical replicates (total *N* = 8).

### 2.10. Statistical Analysis

Parasitemia values on different days were analyzed by the Kruskal—Wallis test. If significant differences (*p* < 0.05) were observed, paired Mann—Whitney U tests were performed to determine which groups were different. Survival curves were analyzed by a log-rank test (Mantel—Cox), using Prism8 and SPSS v.25. The non-parametric Kruskal—Wallis test was performed to determine if the number of amastigote nests was different among the three isolates in each organ analyzed.

## 3. Results

### 3.1. Parasitemia Elicited by Isolates Obtained from Meccus phyllosoma

**Figure 2:**
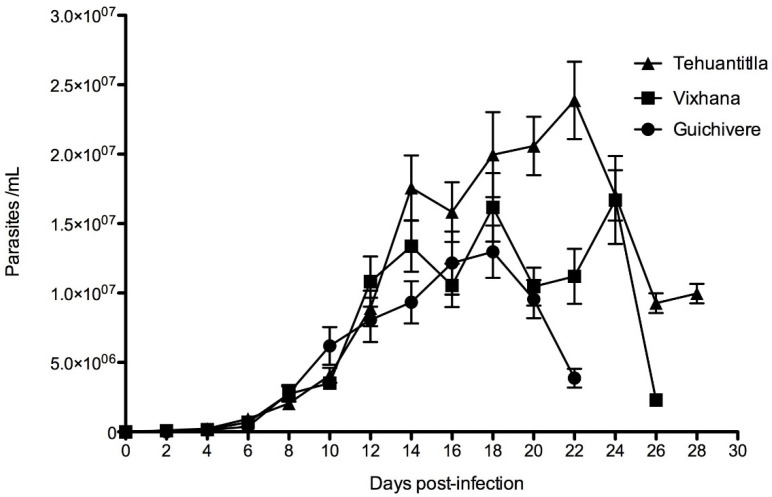
Parasitemia levels in CD-1 mice infected with *T. cruzi* isolates of the state of Oaxaca, Mexico. The isolates of Tehuantitla (**triangle**), Vixhana (**square**), and Guichivere (**circle**) were obtained from the vector *Meccus phyllosoma.* Parasitemia curves were monitored over 30 days post-infection (*N* = 32). Values are expressed as the mean ± standard error.

In the three isolates analyzed herein, blood trypomastigotes (pre-patent period) were detected on the second-day post-inoculation. The Tehuantitla isolates caused a maximum parasitemia of 2.3 × 10^7^ parasites/mL on day 22 post-inoculation, while the Vixhana isolates elicited a parasitemia of 1.7 × 10^7^ parasites/mL on day 24, and Guichivere isolates showed 1.2 × 10^7^ parasites/mL on day 18; the latter isolate had the lowest parasitemia values. Significant differences were found between Vixhana and Guichivere (*p* = 0.028) and Tehuantitla and Guichivere (*p* = 0.002) on day 14. On day 20, significant differences were found between Tehuantitla and Guichivere (*p* = 0.016). Finally, on day 22, significant differences in parasitemia values were also found between Tehuantitla and Guichivere (*p* = 0.028) (Figure 2).

### 3.2. Survival in T. cruzi-Infected Mice

**Figure 3:**
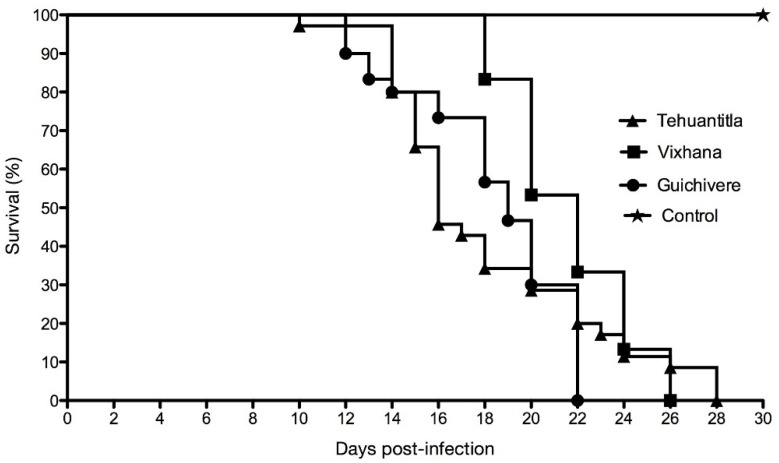
Survival of CD-1 mice infected with Tehuantitla, Guichivere, Vixhana isolates (*N* = 32 in each group), and the control group (*N* = 16) for 30 days post-infection.

The survival of mice infected with *T. cruzi* reached 0% on day 22, 26, and 28 post-infection for the Guichivere, Vixhana, and Tehuantitla isolate, respectively (Figure 3). In contrast, 100% survival was observed in the control group up to day 30. Survival rates were different for mice infected with the three isolates and the control group (log-rank, Mantel—Cox, *χ*^2^ = 92.32, *p* < 0.001). Paired comparisons between the control group and the groups infected with the three isolates showed significant differences (*p* < 0.001 in all cases). However, paired comparisons in survival rates between the three isolates showed no significant differences (*p* > 0.05, in all cases). The median survival time (MST) post-infection for the Tehuantitla isolate was day 16; Vixhana, day 22; Guichivere, day 19.

### 3.3. Identifying and Counting Amastigotes in Organs

**Figure 4:**
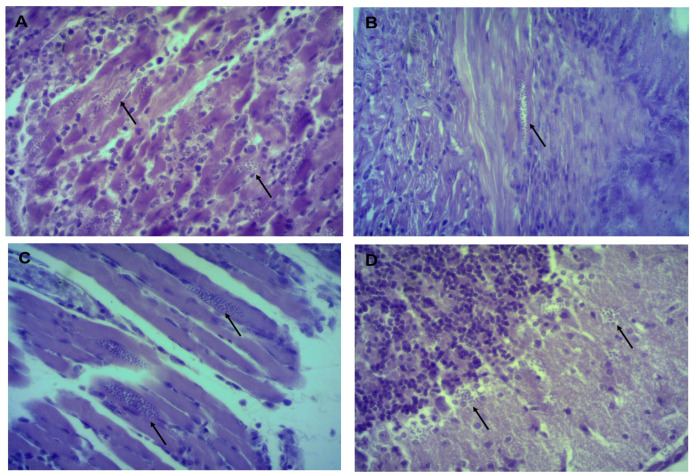
Histological sections of mice infected with the Tehuantitla isolate. Mice were inoculated with 10^6^ parasites/mL and euthanized on the day of maximum parasitemia. Heart (**A**), esophagus (**B**), gastrocnemius (**C**), and brain (**D**). The presence of amastigote nests (arrows) is shown; 40×, light microscopy.

**Figure 5:**
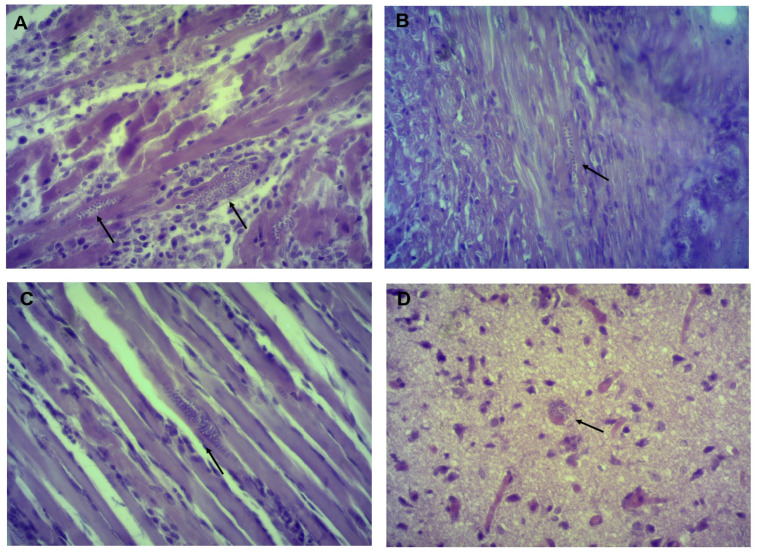
Histological sections of mice infected with the Vixhana isolate. Mice were inoculated with 10^6^ parasites/mL and euthanized on the day of maximum parasitemia. Heart (**A**), esophagus (**B**), gastrocnemius (**C**), and brain (**D**). The presence of amastigote nests (arrows) is shown; 40×, light microscopy.

**Figure 6:**
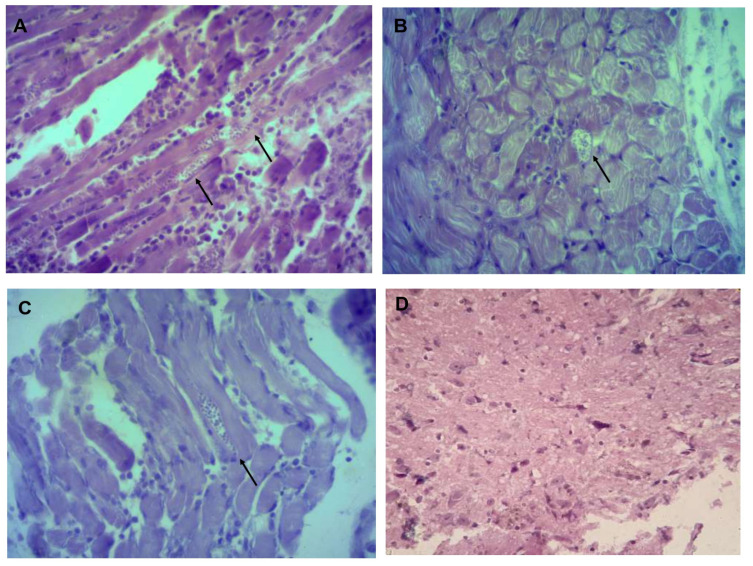
Histological sections of mice infected with the Guichivere isolate. Mice were inoculated with 10^6^ parasites/mL and euthanized on the day of maximum parasitemia. Heart (**A**), esophagus (**B**), gastrocnemius (**C**) and brain (**D**). The presence of amastigote nests (arrows) is shown; 40×, light microscopy.

Histological sections in organs from infected mice showed the presence of amastigotes in heart, esophagus and gastrocnemius in all three isolates, in the brain only in Tehuantitla and Vixhana, along with inflammatory infiltrate (Figure 4, Figure 5 and Figure 6).

**Figure 7:**
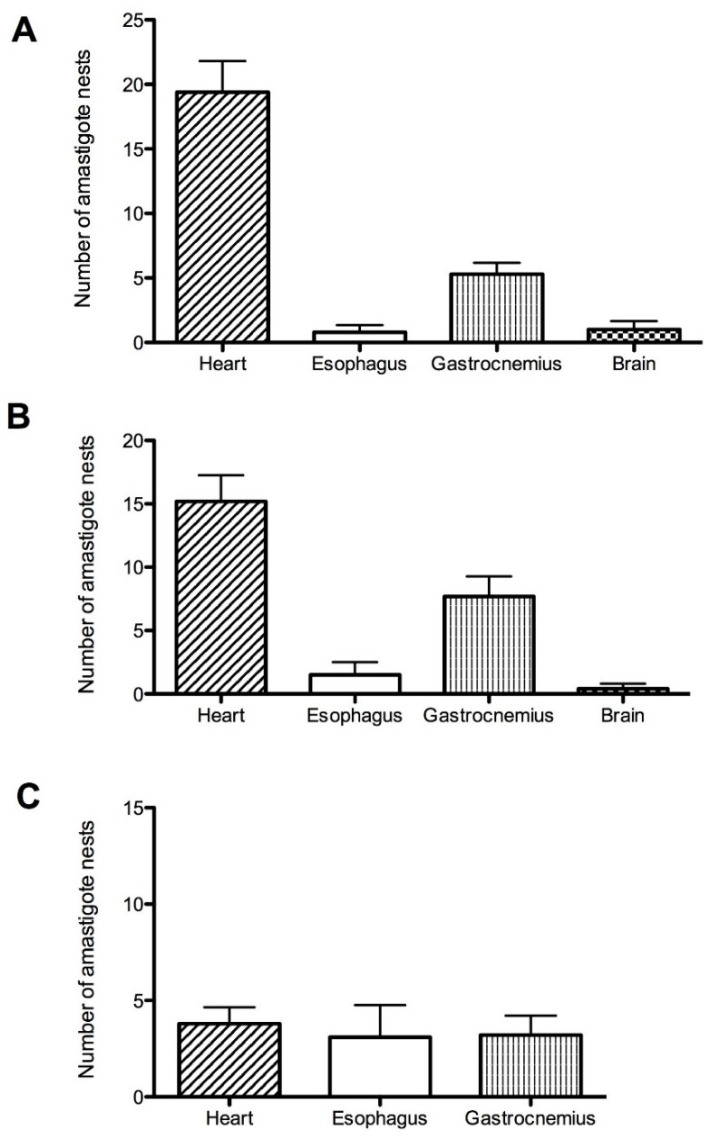
Number of amastigote nests in the Tehuantitla (**A**), Vixhana (**B**), and Guichivere (**C**) isolates. The maximum number of amastigote nests found was *N* = 194, and it was taken as 100%. Values are expressed as the mean ± standard error.

The Tehuantitla isolates yielded the highest number of amastigote nests in the heart (*N* = 194), and this value was taken as 100%; the Vixhana isolates showed 78% of amastigote nests, and the Guichivere isolate showed 20%. In the esophagus, Tehuantitla presented 4%, Vixhana 7%, and Guichivere 16% amastigote nests. Amastigote nests in the gastrocnemius were 27% (Tehuantitla), 40% (Vixhana), and 16% (Guichivere). The amastigote nests in the brain were 5% (Tehuantitla) 2% (Vixhana). No amastigote nests were found in the brain in the Guichivere isolate (Figure 7). Significant differences were found in the number of amastigote nests between the three isolates when the heart was analyzed (Kruskal-Wallis test = 18.642, *p* < 0.001). The Bonferroni post-hoc test showed pairwise differences between Guichivere with Vixhana (*p* < 0.001) and Guichivere with Tehuantitla (*p* < 0.001), with no differences between Vixhana and Tehuantitla (*p* = 0.371). No significant differences were found in any organ in all other comparisons between isolates (*p* > 0.05, in all cases).

### 3.4. Phylogenetic Identification of T. cruzi

**Figure 8:**
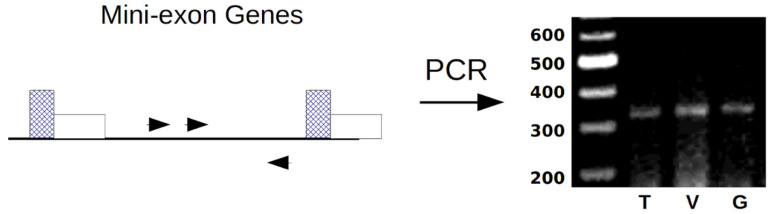
Typing of *T. cruzi* isolates from Oaxaca. Left panel: schematic representation of the *T. cruzi* mini-exon gene PCR typing assay. Perpendicular rectangles with weft represent exonic regions, while horizontal rectangles represent intronic sequences. Oligonucleotides used for the PCR are indicated by arrowheads in the intergenic region (**black line**). Right panel: the PCR-amplified fragments of the intergenic region of mini-exon gene from genomic DNA according to the isolates of Tehuantitla (T), Vixhana (V), and Guichivere (G). Molecular length standards are indicated (200–600 bp). Isolates were classified as TcI, according to the patterns obtained (350 bp).

*T. cruzi* has been classified into six DTUs (TcI, TcII, TcIII, TcIV, TcV, and TcVI) due to its high genetic variability. The intergenic region of the mini-exon gene is widely used to classify *T. cruzi* to isolates by the PCR analysis as follows: 350-bp (TcI) or 300-bp (TcII, TcV, and TcVI), or no product (TcIII and TcIV). In order to genotype and classify each *T. cruzi* isolate from Tehuantitla, Vixhana, and Guichivere, we amplify the intergenic region of the mini-exon gene. The PCR analysis revealed a band of 350 bp in the three isolates, which were thus identified as TcI (Figure 8).

### 3.5. Expression of the T. cruzi Tssa Gene Reveals a Differential Tissue Tropism in the Isolates

**Figure 9:**
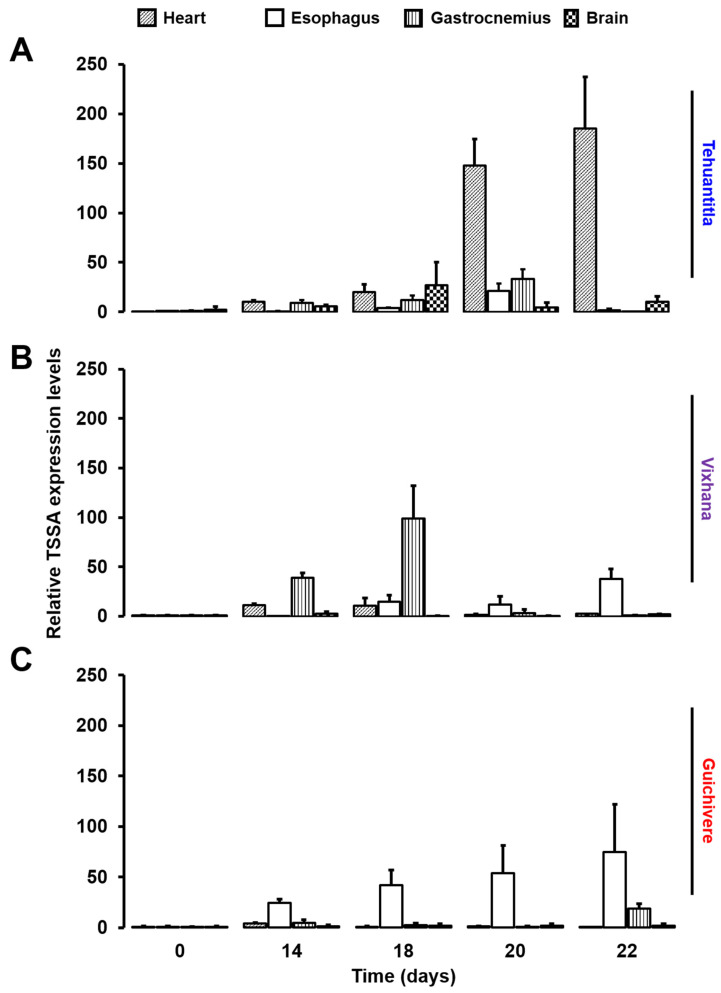
Relative expression levels of the *tssa* gene in different organs of mice infected with *T. cruzi* isolates from different localities in Oaxaca. A qPCR was performed with total RNA from the heart, esophagus, gastrocnemius, and brain of healthy (time 0) and infected (14, 18, 20, and 22 days, post-infection) mice with isolates from Tehuantitla (**A**), Vixhana (**B**), or Guichivere (**C**). The *tssa* expression data were normalized against the expression of the β-actin (*actb*) gene. Error bars indicate the standard deviation (SD) of at least two biological replicates and four technical replicates (*N* = 8).

The presence of *T. cruzi* in the heart, esophagus, gastrocnemius, and brain was determined by detecting changes in the expression of TSSA, a protein coded by the *tssa* gene and expressed on the surface of *T. cruzi* during the infective phase. The total RNA was extracted from each tissue, and the change in the *tssa* expression was analyzed by qPCR. The relative expression levels of the *tssa* in the heart, esophagus, gastrocnemius, and brain on days 14, 18, 20, and 22 post-infection with each isolate are shown in Figure 9. For the Tehuantitla isolate, the *tssa* expression was detected in all four organs on different days post-infection; however, the *tssa* expression predominated in the heart on days 20 and 22 (Figure 9A), also in the brain at day 18, whereas the Vixhana and Guichivere isolates showed an increased expression of the *tssa* in the gastrocnemius and/or the esophagus throughout the infection (Figure 9B,C).

## 4. Discussion

Although all isolates were obtained from the same vector (*M. phyllosoma*), parasitemia values in mice infected with an initial inoculum of 10^6^ parasites/mL differed throughout the infection. The Tehuantitla isolate showed the highest parasitemia, with 2.3 × 10^7^ parasites/mL on day 22, followed by Vixhana, with 1.7 × 10^7^ parasites/mL on day 24, and the lowest parasitemia was elicited by the Guichivere isolate, with 1.2 × 10^7^ parasites/mL on day 22 post-inoculation.

The isolates obtained from other triatomine species have shown different degrees of parasitemia. For example, the isolates obtained from *T. barberi* showed a maximum parasitemia of 21 × 10^6^ parasites/mL on day 6 post-infection, and total mortality occurred at 8 days, while with isolates obtained from *M. pallidipennis*, 1.08 × 10^6^ parasites/mL were counted on day 37 post-infection, and mortality occurred after 59 days [24,25].

Mexican isolates obtained from three species of the genus *Meccus* (*M. pallidipennis*, *M. longipennis*, and *M. picturatus*) from the state of Jalisco, showed maximum parasitemia values of 4.6 × 10^6^ parasites/mL on day 27 post-infection and 10^7^ parasites/mL on day 31; in *M. picturatus*, a sub-patent parasitemia was detected that persisted until 50 days post-infection [26].

Those results suggest that the vector species impacts parasitemia levels and the virulence of isolates. In this study: all three isolates caused 0% survival (Figure 3): However, the lowest survival in the shortest time was observed with the Guichivere isolate, which reached 0% on day 22, in contrast to the Tehuantitla isolate, for which it occurred on day 28, and Vixhana, on day 26.

To our knowledge, no studies have been published on parasitemia and survival in mice inoculated with isolates obtained from *M. phyllosoma*, an endemic vector of epidemiological importance in the region of the Isthmus of Tehuantepec, Oaxaca, whose prevalence could increase given its ability to form hybrids and localize in sympatry with other species. The biological importance of hybrid species (“heterosis” or hybrid vigor) lies in the fact that they have shorter life cycles, more significant number of females (2:1 female to male ratio), high fecundity, and a significant percentage of egg hatching; they are also associated with high entomological indices and resistance to insecticides [6,13].

The isolates studied herein were obtained from vector specimens caught at different localities, all of which were at different altitudes, meters above sea level (masl): Tehuantitla (50 masl), Vixhana (43 masl), and Guichivere (33 masl). As shown in Figure 2, the Tehuantitla isolate elicited the highest parasitemia. In this regard, some authors have mentioned that *T. cruzi* infection rates decrease with altitude in vectors like *M. phyllosoma*, while other authors even indicate that triatomines collected at altitudes above 800 masl do not harbor the parasite [27]. In *T. dimidiata*, it has been shown that altitude influences parasitemia; for example, at 700 masl, parasitemia was 22.57 × 10^6^ parasites/mL; meanwhile, at 300 masl it was 15.66 × 10^6^ parasites/mL, and at 1400 masl it was 11.17 × 10^6^ parasites/mL [16]. In *T. barberi*, it has been reported that, at altitudes of 2030 masl, parasitemia was 21 × 10^6^ parasites/mL [5,17,28]. The behavior and the isolates’ virulence could be influenced by factors underlying *Trypanosoma cruzi*-triatominae interactions, such as temperature, blood source, and immune response and gut microbiota in triatomines [29,30]. On the other hand, the genetic diversity of the parasite and its reproduction by clones could lead to biological differences in virulence and differential tropism in host tissues; in addition, the domestic, peridomestic and wild cycles of natural infection of the insects can influence the behavior of the isolates [14,31].

It is crucial to know the biological behavior of isolates and the heterogeneity of the parasite, as they could correlate with the clinical manifestations in the acute and chronic phases of Chagas disease in humans. In general, damage to the cardiac muscle has been described to occur in the chronic phase of a *T. cruzi* infection, although effects on the digestive tract (megaesophagus or megacolon) and alterations in the autonomic nervous system have also been reported. In Mexico, tropism studies by histology have reported mainly cardiomyopathies, with relatively minor incidences of megaesophagus and megacolon [32].

Histopathological studies have reported amastigote nests in different organs of mice infected with *T. cruzi* isolates. For example, with isolates derived from *T. barberi* (Oaxaca) and *T. mazzottii* (Jalisco), amastigote nests were mostly found in mouse heart and muscle [17,28]. Herein, amastigote nests were identified in mouse heart, esophagus, gastrocnemius (muscle) and brain (Figure 4, Figure 5 and Figure 6). There are few reports of the presence of amastigote nests in brain and esophagus, since the amount is lower than in the heart and its detection is often difficult [33]. In this sense, molecular tests like a qPCR can be very useful to detect low levels of tropism in some organs, as its sensitivity allows us to quantify RNA and DNA of some parasites.

Our genotyping results showed that all *M. phyllosoma*-derived isolates correspond to TcI populations, characterized by a 350 bp amplification band for the mini-exon gene. The TcI populations are classified into domestic (TcIDom) and wild (TcISylv) genotypes, according to their transmission cycle. Infections by this TcIDom occur mainly in northern South America, Central America, and Mexico. These isolates have been predominantly associated with cases of cardiomyopathy and are more resistant to benznidazole than other genetic groups [14,34,35]. In Mexico, it has been shown that isolates obtained in Querétaro and Ninoa belonging to the TcI genotype can invade the digestive system, in addition to causing damage to the heart [33]. Although the TcI lineage predominates in vectors, humans, and wild mammals in Mexico [36], recent studies demonstrated the circulation of TcII, TcIII, TcIV, and TcV genotypes in *T. dimidiata* in the state of Veracruz [37], suggesting a greater diversity of *T. cruzi* DTU in Mexico than previously believed.

The TSSA is a 14-kDa surface antigen mainly expressed at the trypomastigote stage, capable of transferring sialic acid from host glycoconjugates to β-galactopyranose residues to parasite mucins, thereby generating a surface negative charge that protects the parasite from the alternate complement pathway and opsonization by antibodies. It is also linked with adhesion and invasion to epithelial, neuronal, and glial cells [35].

Proteins of this family are also expressed in epimastigotes to promote their adhesion to endothelial cells, in addition to protecting the parasite from the action of glycolytic enzymes in the vector’s digestive tract. At a transcriptional level, the *tssa* mRNA can be detected in trypomastigotes, amastigotes, and epimastigotes [23,35,38]. In patients with a positive history of parasitemia, the *tssa* has a good diagnostic potential for the chronic stage of the disease, where it is found in amastigotes [39].

According to the changes in the expression of the *tssa*, none of the three obtained from *M. phyllosoma* showed organ-specific tropism (Figure 9). However, some trends in the expression of this gene were noted among isolates. The Tehuantitla isolate was detected mainly in the heart, in agreement with the tropism reported for the TcI isolates [14]; this isolate was also detected in the brain at 18-days post-infection. The Vixhana isolate was detected mainly in the gastrocnemius, in mice infected with the Guichivere isolate; the *tssa* expression was detected predominantly in the esophagus. As the disease progresses, *T. cruzi* can invade any cell of the organism except for erythrocytes, although it has been reported to show a marked tropism for neurons and muscle cells [17,29].

The infection with the Tehuantitla isolate resulted in the highest parasitemia levels (Figure 1), lowest median survival time (16 days), and the highest count of amastigote nests in the heart. On the other hand, no statistical difference in survival/mortality was observed, with respect to the Vixhana and Guichivere isolates, and the *tssa* expression was detected mainly in the heart and brain (Figure 9A). Therefore, it is considered to be the most virulent isolate. 

## 5. Conclusions

Taken together, the results of the qPCR analyses suggest that isolates from Tehuantitla, Vixhana, and Guichivere exhibit different tissue tropism: Tehuantitla with predominance for heart and brain, Vixhana for gastrocnemius, and Guichivere for esophagus. No previous studies have reported that the biological behavior of the isolates has correlated parasitemia with the expression levels of the *tssa* gene in different organs.

## Figures and Tables

**Table 1:** Nomenclature and biological/geographical characteristics of the isolates obtained.

Nomenclature	Triatomine Species and Sex	Geographic Area
Tehuantitla (ITRI/MX/2017/TEHUANTITLA)	*M. phyllosoma* Female	Tehuantitla, Tehuantepec municipality, Oaxaca 50 masl Latitude 16°19′06″ N Longitude 95°14′52″ W
Vixhana (ITRI/MX/2017/VIXHANA)	*M. phyllosoma* Female	Vixhana, Tehuantepec municipality, Oaxaca 43 masl Latitude 16°19′06″ N Longitude 95°14′52″ W
Guichivere (ITRI/MX/2017/GUICHIVERE)	*M. phyllosoma* Male	Guichivere, Tehuantepec municipality, Oaxaca 33 masl Latitude 16°19′06″ N Longitude 95°14′52″ W

## Data Availability

The data can be made available upon request to the authors.
